# Ultrastructure and optics of the prism‐like petal epidermal cells of *Eschscholzia californica* (California poppy)

**DOI:** 10.1111/nph.15229

**Published:** 2018-06-01

**Authors:** Bodo D. Wilts, Paula J. Rudall, Edwige Moyroud, Tom Gregory, Yu Ogawa, Silvia Vignolini, Ullrich Steiner, Beverley J. Glover

**Affiliations:** ^1^ Adolphe Merkle Institute University of Fribourg Chemin des Verdiers 4 CH‐1700 Fribourg Switzerland; ^2^ Cavendish Laboratory Department of Physics University of Cambridge JJ Thomson Avenue Cambridge CB3 0HE UK; ^3^ Royal Botanic Gardens Kew Richmond TW9 3AB UK; ^4^ Department of Plant Sciences University of Cambridge Downing Street Cambridge CB2 3EA UK; ^5^ The Sainsbury Laboratory University of Cambridge Bateman Street Cambridge CB2 1LR UK; ^6^ UCL Institute of Archaeology 31–34 Gordon Square London WC1H 0PY UK; ^7^ Department of Chemistry University of Cambridge Lensfield Road Cambridge CB2 1EW UK

**Keywords:** carotenoids, cell wall, flower petals, light scattering, prism

## Abstract

- The petals of *Eschscholzia californica* (California poppy) are robust, pliable and typically coloured intensely orange or yellow owing to the presence of carotenoid pigments; they are also highly reflective at certain angles, producing a silky effect.
- To understand the mechanisms behind colour enhancement and reflectivity in California poppy, which represents a model species among early‐divergent eudicots, we explored the development, ultrastructure, pigment composition and optical properties of the petals using light microscopy and electron microscopy combined with both spectrophotometry and goniometry.
- The elongated petal epidermal cells each possess a densely thickened prism‐like ridge that is composed primarily of cell wall. The surface ridges strongly focus incident light onto the pigments, which are located in plastids at the cell base.
- Our results indicate that this highly unusual, deeply ridged surface structure not only enhances the deep colour response in this desert species, but also results in strongly angle‐dependent ‘silky’ reflectivity that is anisotropic and mostly directional.

## Introduction

Many flowering plants attract pollinators using diverse displays of coloured flowers (Waser, 2006; Whitney & Glover, 2007). Petal colour is typically determined by spectral filtering as a result of wavelength‐selective absorbing pigments present in the petal (Endress, 2001; Vignolini *et al*., 2012a). However, in some species, micro‐ and/or nanostructuring of the petal surface can also result in visible and measurable structural effects. For example, many flowers (e.g. *Antirrhinum majus*) have conical cells on the petal surface that help to focus light into the pigment‐containing vacuole, increase scattering of reflected light and enhance pollinator attraction (Kay *et al*., 1981; Gorton & Vogelmann, 1996; Whitney *et al*., 2011). Conversely, a flat petal surface (e.g. in *Ophrys speculum*) is highly reflective, resulting in a mirror‐like effect (Vignolini *et al*., 2012a,2012b; van der Kooi *et al*., 2017). In other species, parallel striations of the cuticle on the petal epidermis can generate angle‐dependent light scattering (Whitney *et al*., 2009; van der Kooi *et al*., 2014; Moyroud *et al*., 2017). Different regions of the petal surface can display different micromorphologies; for example, in *Hibiscus trionum*, the white region of the petal epidermis consists of smooth conical‐papillate cells while the red‐pigmented region has flat cells with parallel striations (Whitney *et al*., 2009; van der Kooi *et al*., 2014; Vignolini *et al*., 2015).

In this paper, we focus on the flowers of California poppy, *Eschscholzia californica* (Papaveraceae), a drought‐tolerant species that is native to the western United States and Mexico but which is now widely cultivated worldwide. Phylogenetic placement of *Eschscholzia* in the early‐divergent eudicot order Ranunculales makes it an emerging model species in comparative evolutionary studies (e.g. Barakat *et al*., 2007; Hidalgo & Gleissberg, 2010; Lange *et al*., 2013; Damerval & Becker, 2017). The large solitary flowers typically bear four petals that are orange or yellow, owing to the presence of carotenoids, although flower colour variants ranging from white to red are common (Becker *et al*., 2005; Barrell *et al*., 2010). Our preliminary observations of this species revealed a deeply ridged petal epidermis in which the outer wall is unevenly thickened, an unusual surface structure that has not previously been reported in detail as petal surfaces are most commonly composed of either flat epidermal cells or vacuolate conical‐papillate cells with isotropically thickened cell walls (Kay *et al*., 1981; Whitney *et al*., 2011; Vignolini *et al*., 2012a). To investigate the mechanisms behind colour enhancement and reflectivity in this species, we present a detailed ultrastructural, developmental and optical study of its unusual surface morphology. We use a combination of approaches to provide new insights into how the cellular structure of its petal epidermis contributes to an unusually intensely coloured flower.

## Materials and Methods

### Plant material

Flowers and buds of *Eschscholzia californica* Cham. were collected from plants growing in the Cambridge University Botanic Garden, Cambridge, UK, and the Royal Botanic Gardens, Kew, UK, between 2013 and 2018.

### Spectrophotometry

An Olympus (Olympus UK, Southend‐on‐Sea, UK) BX51 light microscope was used for spectroscopic investigations, that is small area microspectrophotometry. Absorbance measurements were performed from petal pieces (average of two to five individual spectra of different coloured areas) that were immersed in a refractive index (RI) matching fluid (Cargille Labs, Cedar Groove, NJ, USA) with RI *n *=* *1.42; the measured transmittance *T*(*λ*) was then converted to absorbance *A*(*λ*) via

$$A\left(\lambda \right)=-\log_{10}\left(T\left(\lambda \right)\right).$$

For UV‐Vis absorbance spectra, the carotenoid pigments of three to five petals were first extracted via ethanol or acetone and the absorbance of these solutions was then measured using a PerkinElmer (London, UK) UV‐Vis spectrometer.

Fibreoptic probe measurements (average of two to three individual spectra of different coloured areas) were performed with a bifurcated reflectance fibreoptic probe (Ocean Optics BIF‐200‐UVVIS; Ocean Optics, Dunedin, FL, USA) using a balanced deuterium‐halogen lamp (Ocean Optics DH‐2000) as light source. Light reflected from the sample was collected through the outer fibres of the probe and analysed using a diode photospectrometer (Ocean Optics QE65000). A diffuse white Lambertian reflectance standard (Spectralon SRM‐99; Labsphere, North Sutton, NH, USA) served as a reference.

### Goniometry

Goniometry measurements were performed with a custom‐built goniometer setup (Vukusic & Stavenga, 2009; Vignolini *et al*., 2013) on fresh petal samples and epoxy casts. Briefly, light from a light source is collimated onto the sample, which is mounted on a rotating goniometer. Centred on this central goniometer is an arm where a second optical fibre is mounted that collects the scattered light and guides it to a diode spectrophotometer. This allows the measurement of diffraction, scattering and reflectance for arbitrary angles of light incidence, *θ*_in_, and detection, *θ*_out_.

### Imaging scatterometry

The far‐field spatial distribution of the light scattered from an epoxy replica of a flower petal was visualized using an imaging scatterometer (Vukusic & Stavenga, 2009; three casts from different petals were analysed). Small samples were mounted on the tip of a micropipette and positioned in the mirror's first focal plane. Light scattered by the sample into the frontal hemisphere is focused by an ellipsoidal mirror in its second focal plane and projected by a lens onto its back focal plane, thereby compressing the far‐field scattering pattern into an image that is captured using a charge‐coupled device camera. Custom‐written Matlab (https://www.mathworks.com) programs correct the resultant scatterograms for aberrations. A piece of MgO served as a white standard.

### Electron microscopy

Scanning electron microscopy (SEM) was performed using a Zeiss Leo Gemini 1530VP FEG‐SEM (for epoxy‐resin samples; Oberkochen, Germany) or a Hitachi S‐4700 SEM (for critical‐point dried plant material; Tokyo, Japan). To prevent charging effects, the samples were sputter‐coated before imaging with a *c*. 2 nm layer of gold or platinum. Cryogenic scanning electron microscopy (cryo‐SEM) observation was performed using a field‐emission scanning electron microscope (Verios 460; ThermoFisher Scientific Inc., Pittsburgh, PA, USA) equipped with a cryopreparation system (PP3010T; Quorum, Laughton, UK). The petal was cut into a small strip and mounted upright on a specimen holder using a colloidal graphite suspension. The specimen was quench‐frozen in liquid ethane and transferred into the cryopreparation chamber, where it was freeze‐fractured, sublimed, and subsequently sputter‐coated with platinum. SEM imaging was carried out at an acceleration voltage of 2 kV and a working distance of *c*. 4 mm.

For examination using transmission electron microscopy (TEM), 2 mm^2^ regions of petal tissue were dissected using a mounted needle, fixed in 2.5% glutaraldehyde in phosphate buffer at pH 7.4, stored in 70% ethanol before being stained in 1% osmium tetroxide solution and passed through an ethanol and resin series before being polymerized for 18 h under vacuum. A Reichert‐Jung (Vienna, Austria) Ultracut microtome was used to cut semithin (0.5–2 μm) and ultrathin (50–90 nm) sections. The semithin sections were mounted on glass slides and stained with toluidine blue in phosphate buffer, before examination under a light microscope. The ultrathin sections were placed on copper mesh grids before imaging with a Hitachi H‐7650 TEM.

### Raman scattering

A Renishaw (Wotton‐under‐Edge, UK) InVia Raman microscope was used for all measurements in point scan mode on the ethanol extracts. A 50× long working‐distance objective was used to collect all spectra. A grating of 1200 lines mm^−1^ was used with an appropriate edge filter. Raman modes were excited with a 633 nm HeNe laser with typical incident power of ≈1 mW.

### Finite‐difference time‐domain modelling

Light scattering by the internal structure of the petals was simulated using a three‐dimensional finite‐difference time‐domain (FDTD) method using the ultrastructure obtained from TEM images. The use of FDTD modelling has a number of advantages over classic modelling methods, including allowing the input of measured TEM images with an assignment of a RI to each greyscale and calculating a spatial light scattering pattern reflected from that structure (Taflove & Hagness, 2005; Wilts *et al*., 2014). For the simulations, we used Lumerical 8.15 (Lumerical Solutions Inc., Vancouver, BC, Canada), a commercial‐grade simulator implementing the FDTD method.

### Floral temperature measurements

Temperature measurements of the base and tip of 11 petals from two plants grown in different glasshouse conditions were taken using a hand‐held infrared thermometer (N19FR InfraRed Thermometer; Maplin, Rotherham, UK).

## Results

### Petal structure and development

**Figure 1:**
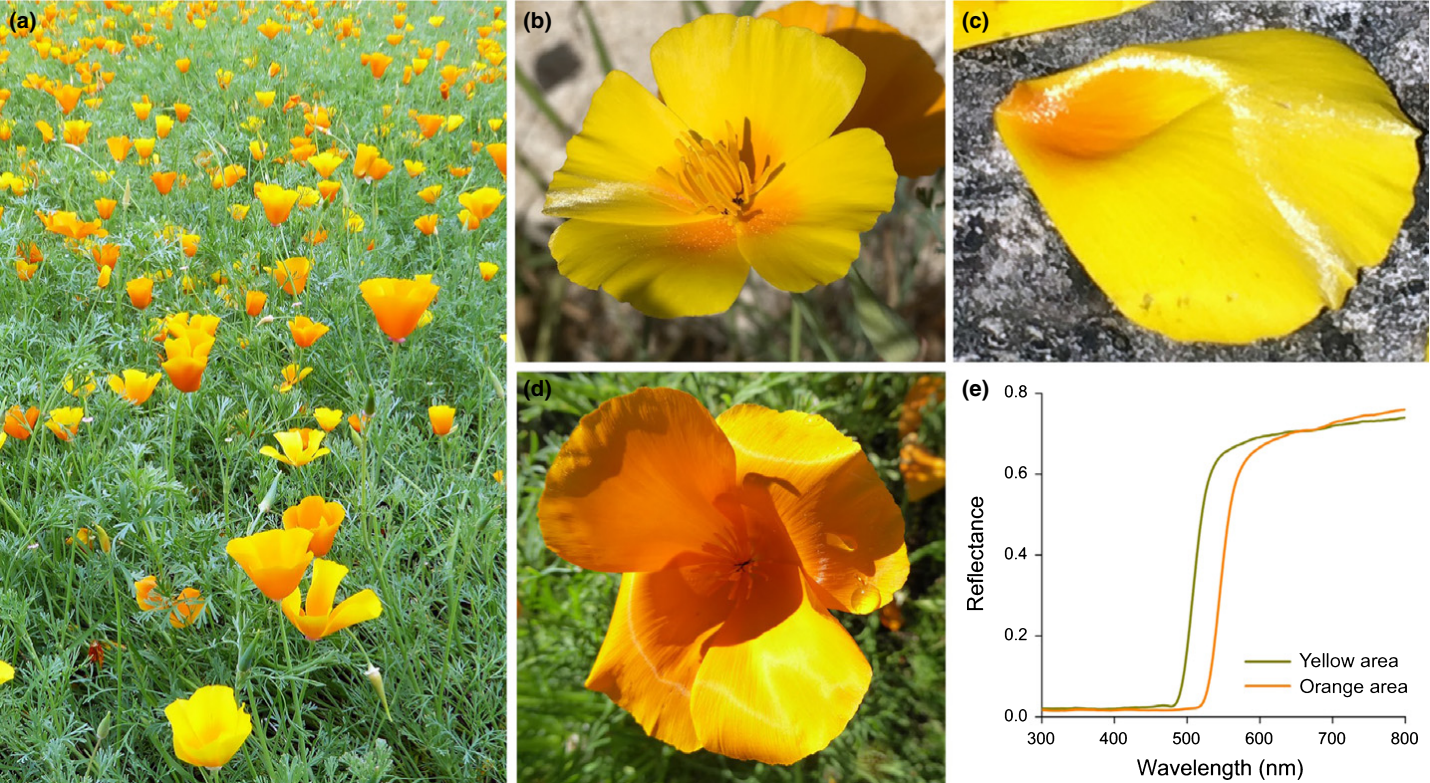
California poppy, *Eschscholzia californica*. (a) A field of *E. californica* in the Royal Botanic Gardens, Kew. (b) Entire flower, with four petals that are yellow with an orange centre. (c) Dissected petal illustrating the gloss effect. (d) Entire orange flower indicating the pliability of the petals. (e) Probe reflectance spectra of the different colours.

**Figure 2:**
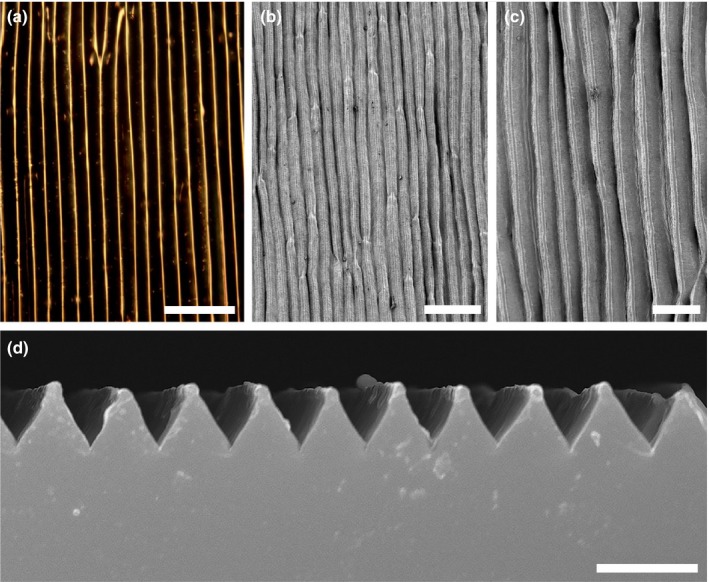
Adaxial surfaces of mature petals. (a) Bright‐field light micrograph showing anticlinal cell walls on the adaxial epidermis of the petals. (b) Scanning electron micrograph showing the strongly ridged structure of the adaxial surface. (c) Detail of surface ridges. (d) Transverse section of an epoxy replica showing regular surface ridges. Bars: (a, c) 20 μm; (b) 50 μm; (d) 10 μm.

Mature petals of *E. californica* are intensely coloured (Fig. 1a–e) on both adaxial and abaxial surfaces and appear unusually pliable. The pliability is probably due, at least in part, to the robustly thickened outer epidermal cell walls, which also results in the appearance of a silky texture (Fig. 1b,c). The reflectance spectra of the orange and yellow regions of the petal show the typical sigmoidal shape of pigmented materials (Fig. 1e). Both petal surfaces show a regular pattern of longitudinally elongated cells (parallel to the proximodistal axis of the petal; Fig. 2) *c*. 65–80 μm in length, each cell bearing a distinct central ridge with a spacing of 10.8 ± 0.4 μm (Table 1). A cross‐section of the cast indicates that these ridges create a petal surface resembling a regular array of prism‐like shapes with a height of *c*. 8 μm (Fig. 2d).

**Table 1:** Developmental series of California poppy – length of investigated buds, *L*_bud_, and average separation distance of ridges, *d*_r_ (*n *= number of ridges measured, ± SEM)

Sample	*L*_bud_ (mm)	*d*_r_ (μm)
Developmental stage 1 (*n *=* *31)	9 ± 2	6.4 ± 0.3
Developmental stage 2 (*n *=* *43)	12 ± 1	6.3 ± 0.3
Developmental stage 3 (*n *=* *47)	17 ± 1	6.1 ± 0.3
Mature petal (*n *=* *76)	—	10.8 ± 0.4

**Figure 3:**
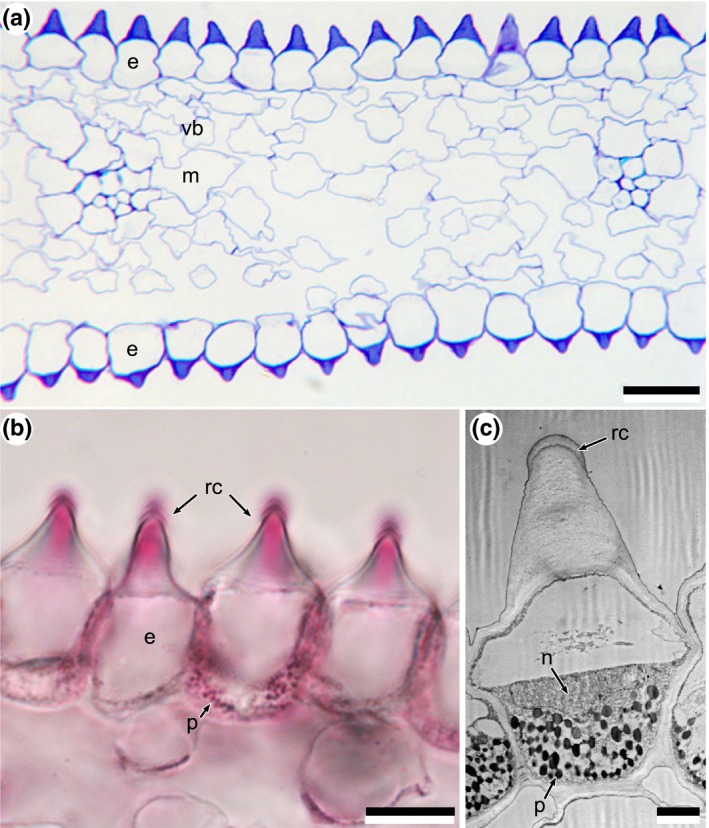
Transverse sections of mature petals. (a) Light micrograph of a transverse section of the entire petal. (b) Part of adaxial epidermis stained with fat red to highlight lipid‐rich regions, indicating that most of the prism is not made of cuticular material. (c) Transmission electron micrograph of an epidermal cell showing cell contents predominantly located at the base of each cell. e, epidermis; m, mesophyll; n, nucleus; p, pigment‐containing plastids; rc, cap (apex) of ridge; vb, vascular bundle. Bars: (a) 20 μm; (b) 10 μm; (c) 2 μm.

**Figure 4:**
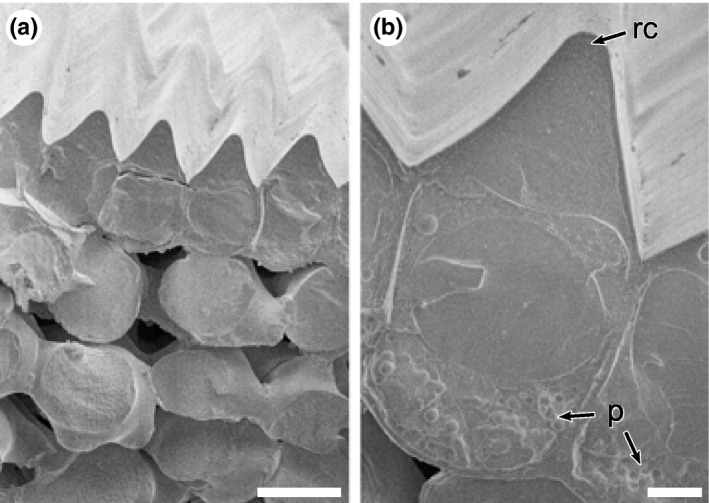
Cryogenic scanning electron microscopy images of mature petal surfaces at different magnifications. (a) View onto a transverse section of a petal showing multiple ridged cells in the adaxial epidermis and mesophyll cells below. (b) High‐magnification image of a single adaxial cell. p, pigment‐containing plastids; rc, cap (apex) of ridge. Bars: (a) 10 μm; (b) 2 μm.

Both the adaxial and abaxial epidermal surfaces exhibit similarly ridged cells, although the ridges are slightly less pronounced on the abaxial surface (Fig. 3a). The ridges are unusual in that they are not vacuolated but instead represent a solid structure that is predominantly composed of cell wall with a cuticular cap on the top of the ridge (Figs 3b,c, 4). The cell content consists of a large vacuole overlying a cytoplasmic region that contains a nucleus and pigment plastids (Fig. 3b,c).

**Figure 5:**
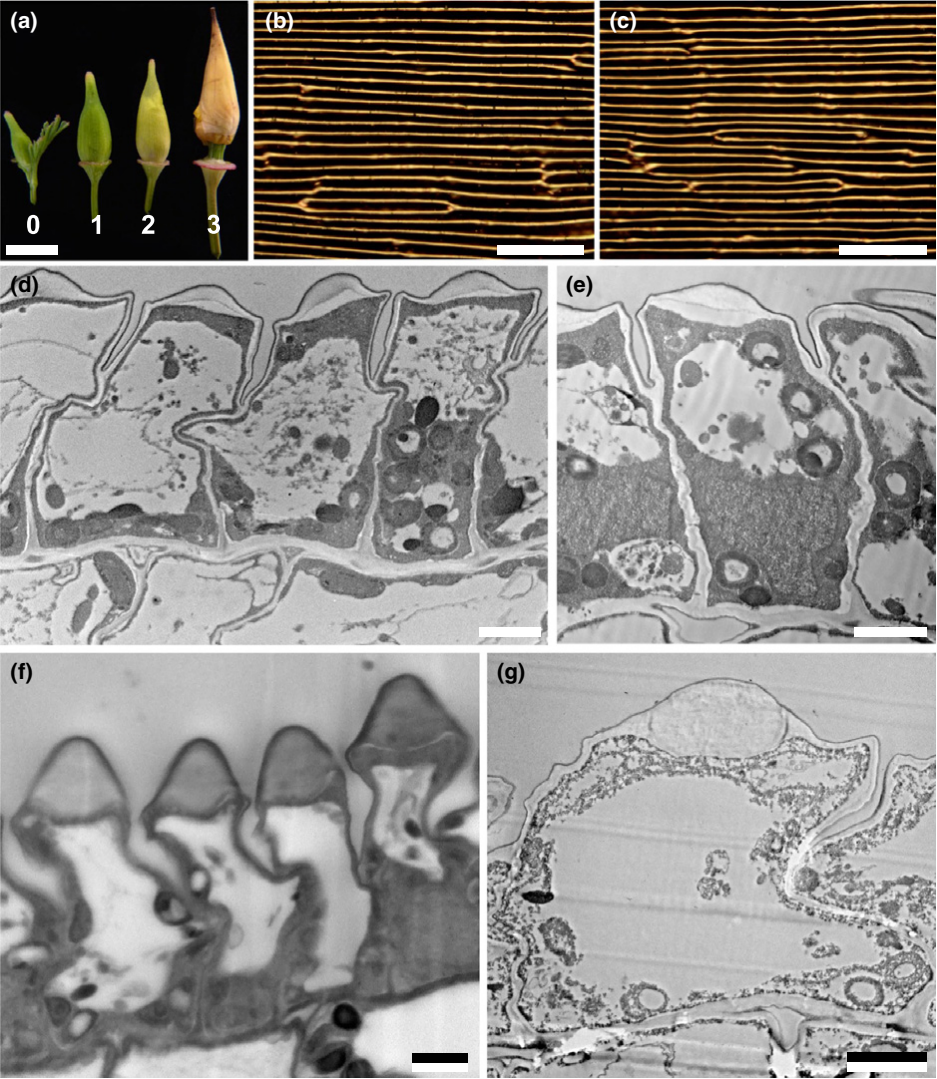
Development of the petal epidermis. (a) Photograph of flower buds at four successive developmental stages (bud lengths: stage 0, 5 mm; stage 1, 9 mm; stage 2, 12 mm; stage 3, 16 mm). Bud stage 0 is equivalent to developmental stage 8 of Becker *et al*. (2005); stages 1–3 are encompassed within developmental stage 9 of Becker *et al*. (2005). (b, c) Bright‐field light micrographs of the adaxial petal surface showing anticlinal cell walls for petals of stages 1 and 3. (d, e) Transmission electron microscopy (TEM) images of stage 1; epidermal cells are anticlinally elongated but the ridges are not well developed; the cell contents are relatively evenly distributed, although a large vacuole is developing. (f, g) TEM images of stage 3; epidermal cells have grown both anticlinally and periclinally and the ridges and central vacuole are developed further. Bars: (a) 5 mm; (b, c) 50 μm; (d–g) 2 μm.

To explore how this unusual petal ultrastructure develops, we investigated the surface structure of petals at four different stages of bud development (Fig. 5). These stages were defined by bud length – our stage 0 corresponds to flower development stage 8 of Becker *et al*. (2005), while our stages 1–3 are encompassed within flower development stage 9 of Becker *et al*. (2005). The ridges start to become visible at our stage 1 when the buds are 9 mm in length. At this stage, the cells are relatively slender, resulting in a narrow spacing between adjacent ridges of 6.4 ± 0.3 μm, compared with 10.8 ± 0.4 μm for mature petals (Table 1). The ridges subsequently extend and become more pronounced as the petal develops.

### Pigment identification

**Figure 6:**
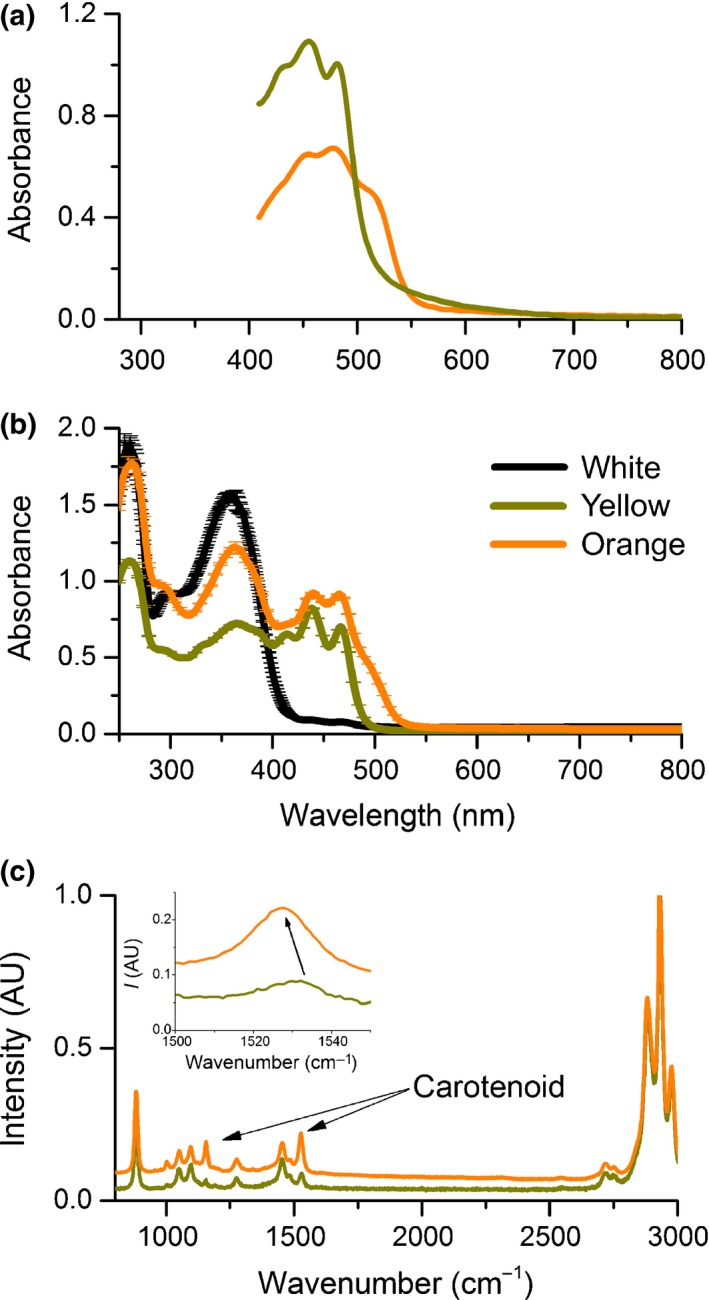
Pigment identification. (a) Absorbance spectra derived from different coloured petal pieces (yellow or orange) immersed in refractive index (RI) oil with *n *=* *1.42. (b) Absorbance spectra taken from ethanol extracts from white, yellow or orange petal pieces. The three‐peaked absorbance spectra are a clear signature of carotenoid pigments. (c) Raman spectra from pigment extracts upon excitation with a 532 nm laser with *c*. 1 mW power. The peaks characteristic for carotenoids are marked. The shift of the Raman peak to shorter wavenumbers for the orange extract is indicative of a longer carotenoid chain.

To identify the absorbing pigments in the petals, pieces sampled from yellow and orange regions of the petal were immersed in RI matching fluid (*n *=* *1.42) and the absorbance spectra were measured with a microspectrophotometer (Fig. 6a). Immersion in a RI fluid suppresses interfacial scattering and thus allows measurement of the spectral characteristics of the absorbing pigments. Both absorbance spectra show a three‐peaked absorbance spectrum, characteristic of carotenoid pigments (Thomas *et al*., 2013). Whereas the orange pigment shows a peak absorbance *in vivo* at *c*. 480 nm (with additional peaks at 429 and 454 nm), the yellow pigment absorbs maximally at *c*. 450 nm (with additional peaks at 413 and 438 nm). Ethanol extracts of various petals confirm the three‐peaked absorbance (Fig. 6b) and resonance Raman spectra (Fig. 6c) confirmed that the pigments are indeed carotenoids (although the peaks are hypsochromically shifted as a result of solvation), as previously investigated in detail (see, e.g. Strain, 1938; Zang *et al*., 1997; Maoka *et al*., 2000; Withnall *et al*., 2003; Schulz *et al*., 2005; Barrell *et al*., 2010).

### Optical properties

**Figure 7:**
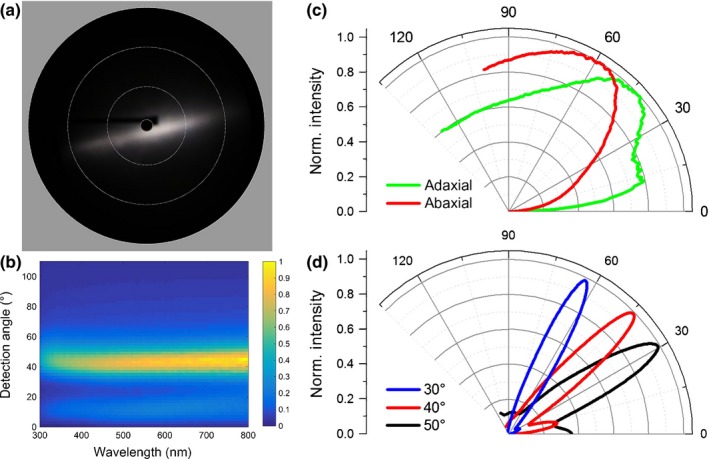
Optics of mature petal of *Eschscholzia californica*. (a) Imaging scatterometry of the epoxy cast corresponding to the adaxial surface. The circles indicate scattering angles of 5°, 30°, 60° and 90°. Light scattering by the cast's surface is highly anisotropic, as shown by the appearance of a line in space – a mirror would, for instance, only reflect a single spot (cf. van der Kooi *et al*., 2017). (b) Goniometry spectra for a fixed angle of incidence of 135° of a fresh petal from the adaxial epidermis. (c) Polar plot of average reflectance in the wavelength range 550–650 nm for a fresh petal from the adaxial (green) and abaxial (red) epidermis. (d) Goniometry of an epoxy replica of the adaxial epidermis for angles of incidence of 30° (blue), 40° (red) and 50° (black). Goniometry results confirm the directional and anisotropic light scattering by the petal.

To investigate the reflective appearance of the petals, we characterized the reflected light scattering using an imaging scatterometer (Stavenga *et al*., 2009) as well as a goniometric measurement setup, which allows both illumination and detection angles to vary (Fig. 7) (Vukusic & Stavenga, 2009; Vignolini *et al*., 2013). Imaging scatterometry of an epoxy replica of the petal eidermis showed that the surface scattering of the petal is directional and limited to a reflection line across the long direction of the petals (Fig. 7a). The goniometric measurements of fresh petals shown in Fig. 7(b,c) were obtained by illuminating the sample at an angle of −45° (or 135°) to the petal normal and then varying the detection angle between 0° and 135°. Note that 45° is the specular reflection direction, where directional reflection occurs. Both the abaxial and adaxial sides of the petal scatter light quite directionally, with maxima at 45° (Fig. 7b,c), and significantly broaden the incident beam. The light reflectance of the surface is strongly angle‐dependent, that is, anisotropic and mostly directional. By comparing the optical signature of the fresh petal with the epoxy replica (Fig. 7c,d), which replicated the surface and not the internal petal structure, a spatial broadening of the angular reflectance profile is observed. It is therefore likely that this broadening arises from scattering of light inside the petal structure, that is, the various compartments of the upper layer and underlying cells, highlighting the role of petal composition throughout the petal thickness.

**Figure 8:**
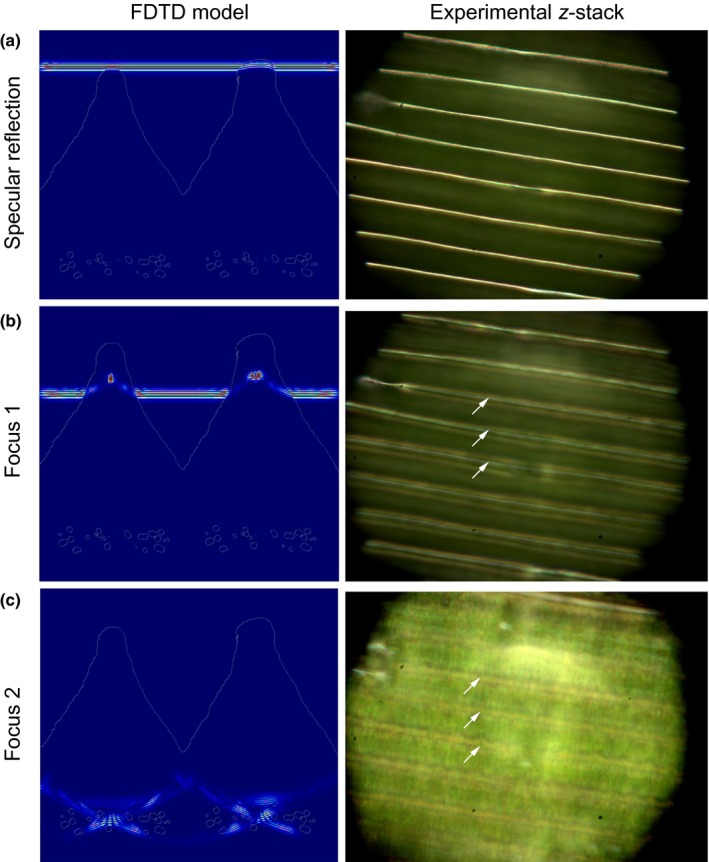
Focusing of light through the epidermal cells of an *Eschscholzia californica* petal. Finite‐difference time‐domain (FDTD) modelling (left column) indicates the presence of three different optical effects at different heights of the petal epidermis: (a) a directional, specular reflection from the petal surface; (b) a first focus caused by the microlens formed by the epidermal cell tip; and (c) a second focus located in the basal part of the cells where the pigment‐containing plastids are localized. A focus stack (*z*‐stack) obtained from optical microscopy (right column) of a mature petal supports these findings and confirms the appearance of three different optical effects. The arrows point to the position of the ridges and highlight that the optical foci are below the ridge surface.

The prism shape of the epidermal cells induces optical focusing within the epidermis. Snapshots of FDTD simulations on the petal ultrastructure gained by TEM show that incident light is focused into two foci within the epidermal prism‐like cell (Fig. 8). Light is first scattered from the top surface of the petal (Fig. 8a, left column), resulting in a striped, line‐like pattern (Figs 2, 3). Light coupled into the petal structure results in a focus within the prism‐shaped structure, caused by local microlensing of the prism cap (Fig. 8b, left column). Significantly, the second focus point coincides with the position of the pigment granules at the base of the cell (Fig. 8c, left column). We performed optical microscopy to confirm these effects on a fresh, mature petal. Indeed, changing the focus (*z*‐stacking) showed the presence of two different foci along the apicobasal axis of the cell (indicated by white arrows in Fig. 8, right side). Varying the RI of the top cap layer within reasonable RI boundaries in the FDTD simulations did not change this result.

### Floral temperature

The reflective surface of *E. californica* could function to enhance temperature in the centre of the flower, potentially benefiting development of gametes and the young embryo (Kevan, 1975). To investigate this possibility, we measured temperature at the tip and base of 11 petals from *E. californica* plants grown in two different temperature regimes. In all cases, the base of the petal (centre of the flower) was either cooler or the same temperature as the tip (Table 2).

**Table 2:** Floral temperature of California Poppy – temperatures measured at the base and tip of *Eschscholzia californica* petals (*n *=* *11) from plants grown in temperate (21°C) and tropical (28°C) glasshouse conditions

Petal sample	Temperature at base (°C)	Temperature at tip (°C)	Plant growth conditions
1	27.1	26.4	Tropical
2	27.6	26.8	Tropical
3	27.9	27.2	Tropical
4	27.3	26.8	Tropical
5	22	21.3	Temperate
6	22.2	21.8	Temperate
7	21.9	21.7	Temperate
8	21.9	21.2	Temperate
9	27.9	27.4	Tropical
10	27.9	27.9	Tropical
11	21.7	21.4	Temperate

## Discussion

### Unusual epidermal structure in *E. californica* petals

Our study demonstrates that the unusual prism‐like shape of the ridged petal epidermal cells of *E. californica* is composed primarily of cell wall. This structure imposes optical properties not previously demonstrated in petals: it focuses light onto carotenoid‐containing plastids at the base of the epidermal cells. This observed prism‐like shape is highly unusual; petal surfaces are most commonly composed of either elongated flat cells or isotropically structured conical‐papillate cells (Kay *et al*., 1981; Whitney *et al*., 2011; Vignolini *et al*., 2012a). The circular or hexagonally based conical‐papillate cells that characterize petals of many other eudicots differ markedly in that the wall is relatively evenly thickened around the entire cell as the conical outgrowth is formed by turgor‐driven expansion of the entire cell and is therefore vacuolated in cross‐section (Noda *et al*., 1994). The petal epidermal cells of *E. californica* apparently represent a unique structure. Among other members of the early‐divergent eudicot order Ranunculales, petal epidermal cells are mostly either conical‐papillate or relatively flat and elongated. Kay *et al*. (1981) described ‘multiple reversed‐papillate’ petal epidermal cells in species of *Chelidonium* and *Papaver* (Papaveraceae – Ranunculales), in which the outer surface is flat but the inner surface is convex. However, our results do not support a similar structure for *Eschscholzia*. Recent observations of *Adonis aestivalis* (Ranunculaceae – Ranunculales) show that the epidermal cells are evenly thickened and shallowly domed, with parallel surface striations (Moyroud *et al*., 2017). Thus, the early‐divergent eudicot order Ranunculales, which is critical in understanding trait evolution in eudicots, displays considerable diversity not only in overall flower structure (Damerval & Becker, 2017), but also in ultrastructural aspects of the petal epidermis.

Our analysis of petal development shows that the pronounced surface ridges are not formed by turgor‐driven expansion of the cell itself, but by successive local deposition of cell wall on the top of the epidermal cell. This cell wall deposition does not occur evenly but accumulates at the midline of the cell to create the characteristic prism shape. At the final maturation step (between our stage 3 ‘16 mm bud’ and ‘mature petal’), the size of the cells and hence the distance between adjacent prism‐like cells almost doubles (Fig 5; Table 1). Carotenoid pigments are already present in the cytoplasm at an early stage of petal development. Thus, the deep colouration is only fully established in a late‐stage preanthetic bud (Fig. 5); at earlier stages, the spacing between the midlines of neighbouring cells is too small to focus the light in the pigment‐rich basal part of the cell.

### Light localization at the pigment‐rich part of the cell enhances flower colour

Localization of floral pigments differs among different plant species, both within the petal itself and within individual cells (Unterlinner *et al*., 1999; Vignolini *et al*., 2012a; van der Kooi *et al*., 2016). Throughout the petal, pigments can be localized in three different ways, each resulting in subtle differences in the overall optical appearance of the petal: the pigment is evenly distributed throughout the entire petal; both adaxial and abaxial epidermal layers are pigmented, whereas the intervening mesophyll layers are unpigmented; the pigment is localized in only one of the two epidermal layers. Within individual cells, different types of pigment may be either contained centrally within the large vacuole or distributed in plastids in the peripheral cytoplasm (Vignolini *et al*., 2012a; van der Kooi *et al*., 2016).

The prism‐like petal epidermis of *E. californica* enhances the bright yellow/orange colour intensity by reflecting light from the carotenoid pigments in the lower part of the cell (Strain, 1938; Maoka *et al*., 2000; Barrell *et al*., 2010). Compared with water‐soluble pigments such as anthocyanins, which normally accumulate in the cell vacuole, carotenoid pigments are contained in plastids, typically located close to the inner periclinal walls of the epidermal cells. This is indeed the case in *E. californica*, in which the carotenoid pigments are localized in plastids at the base of the epidermal cells, below the vacuole and at the opposite pole to the prism‐like outer cell wall. This optical system focuses light strongly onto the pigment‐containing region (Figs 7, 8) and results in optimized light extraction from the pigments positioned at the base of the cell. Our results indicate that this system allows a pigment localized in a limited spatial region to achieve a colour effect similar to that seen in homogenously filled cells, while at the same time limiting the spatial width of the reflected signal, resulting in the silk‐like texture of the petal. The pigments in the plastids must be photostable to provide a constant optical appearance throughout the lifetime of the matured cell, a property known particularly for carotenoids present in the plastoglobuli of chloroplasts (Solovchenko, 2010).

### A mechanism to produce ‘silky’ reflectivity

Many flowers feature a distinct ‘shine’ overlying their pigmentary coloration (Fig. 1). Our results indicate that more rigorous terminology is required to differentiate between the different types of highly reflective surfaces that occur on petals of some species. The mirror‐like (glossy) surface of the petals of the buttercup, *Ranunculus* (Parkin, 1928; Galsterer *et al*., 1999; Vignolini *et al*., 2012b; van der Kooi *et al*., 2017), and the speculum of the mirror orchid, *Ophrys speculum* (Vignolini *et al*., 2012a), are the result of a very flat petal surface with smooth epidermal cells that reflect incident light. This surface reflects light mainly in the specular reflection direction and appears similar to a rigid glossy surface. By contrast, in the California Poppy, *E. californica*, the local structuring of the epidermis (Fig. 4a) gives rise to a different visual effect on the petal, to produce the observed reflectivity we term ‘silky’. We use the term ‘silky’ as the petal looks exceptionally similar to a glossy nonrigid surface reminiscent of some glossy textiles. This term is consistent with a softer appearance with less uniform reflectivity. The unusual silky effect of *E. californica* is produced by the intense angle‐dependent reflectivity from the deeply ridged petal epidermal cells, which is anisotropic and mostly directional, as well as the focusing effect of the prism‐shaped ridges (Figs 7, 8).

The biological significance, if any, of such a silky surface remains to be investigated in depth. However, a highly reflective appearance could potentially have two effects, which may even operate synergistically. The first of these is the creation of an appealing and recognizable visual signal to pollinators that contrasts with a more ‘dull’ background. A similar function has been described for the conical cells that focus light into anthocyanic vacuoles in *Antirrhinum majus*. Glover & Martin (1998) demonstrated that *A. majus* plants with conical petal cells were visited more frequently by foraging bees than otherwise isogenic plants that lack the conical petal cell morphology. In a later study with the same lines, Dyer *et al*. (2007) demonstrated that the colour enhancement provided by the conical cells relative to the isogenic flat‐celled line was visible to *Bombus terrestris* foragers. Similarly, a study in Greater Spearwort (*Ranunculus lingua*) demonstrated that petal gloss could increase flower conspicuousness to pollinators by providing a dynamic visual display (Galsterer *et al*., 1999).

The second potential effect of the silky reflectivity is the warming of the flower, as a result of energy incoupling to the reproductive organs at the centre, possibly speeding up seed development (Kevan, 1975). This possibility has also been proposed for glossy buttercups (van der Kooi *et al*., 2017), which have previously been shown to be warmer at the centre of the flower (Cooley, 1995). Synergy between the attraction of pollinators and the warming of the flower might also be possible, as previous studies have shown that bumblebees prefer warmer flowers (Dyer *et al*., 2006). However, our measurements indicate that the centre of the *E. californica* flower is not warmer than the tips of the petals, and may even be cooler, ruling out a role in intrafloral warming for this particular petal epidermal geometry. The full role of silky reflectivity in mediating the communication between flowering plants and their animal pollinators remains to be tested, and will require field experiments in the appropriate ecological context with plants differing only in reflectivity traits.

### Conclusions

Our study in *E. californica* describes an unusual type of petal epidermal ultrastructure which has apparently not been previously reported in flowering plants. In contrast with the domed or conical, evenly thickened cell walls of the petals of many other species, those of *E. californica* possess dense thickened prism‐like ridges that promote the strong colour intensity that potentially enhances their attraction to pollinators but that also increase angle‐dependent reflectivity. High colour and reflectivity are traits often associated with drought‐tolerant plants, and this likely correlation requires more detailed investigation. Despite considerable ongoing interest in floral organ identity in early‐divergent eudicots such as *Eschscholzia* (e.g. Lange *et al*., 2013), little is currently known about the developmental genetics of flower colour in this genus. Our results highlight the diverse range of strategies adopted by petals of different species to enhance colour and reflectivity, even among relatively closely related species. Future comparative studies will focus on the diversity of petal form and reflectivity within a single order, Ranunculales.

## Author contributions

B.D.W. performed the spectrophotometry, goniometry analyses and FDTD modelling. P.J.R., E.M., T.G. and Y.O. conducted the transmission and (cryo‐) scanning electron microscopy and cuticle staining. E.M. and B.J.G performed the temperature measurements. B.D.W., P.J.R., E.M., S.V., U.S. and B.J.G. designed the project and wrote the manuscript.
